# Trends in low-value GP care during the COVID-19 pandemic: a retrospective cohort study

**DOI:** 10.1186/s12875-024-02306-7

**Published:** 2024-02-28

**Authors:** Joris L. J. M. Müskens, Tim C. Olde Hartman, Henk J. Schers, Reinier P. Akkermans, Gert P. Westert, Rudolf B. Kool, Simone A. van Dulmen

**Affiliations:** 1 Radboud University Medical Center, IQ Health Science Department, Nijmegen, The Netherlands; 2 Radboud University Medical Center, Department of Primary and Community Care at Radboud Institute for Health Sciences, Nijmegen, the Netherlands

**Keywords:** COVID-19, Low-value care, Quality of Health Care, General practice

## Abstract

**Background:**

Several studies showed that during the pandemic patients have refrained from visiting their general practitioner (GP). This resulted in medical care being delayed, postponed or completely forgone. The provision of low-value care, i.e. care which offers no net benefit for the patient, also could have been affected. We therefore assessed the impact of the COVID-19 restrictions on three types of low-value GP care: 1) imaging for back or knee problems, 2) antibiotics for otitis media acuta (OMA), and 3) repeated opioid prescriptions, without a prior GP visit.

**Methods:**

We performed a retrospective cohort study using registration data from GPs part of an academic GP network over the period 2017–2022. The COVID-19 period was defined as the period between April 2020 to December 2021. The periods before (January 2017 to April 2020) and after the COVID-19 period (January 2022 to December 2022) are the pre- and post-restrictions periods. The three clinical practices examined were selected by two practicing GPs from a top 30 of recommendations originating from the Dutch GP guidelines, based on their perceived prevalence and relevance in practice (van Dulmen et al., BMC Primary Care 23:141, 2022). Multilevel Poisson regression models were built to examine changes in the incidence rates (IR) of both registered episodes and episodes receiving low-value treatment.

**Results:**

During the COVID-19 restrictions period, the IRs of episodes of all three types of GP care decreased significantly. The IR of episodes of back or knee pain decreased by 12%, OMA episodes by 54% and opioid prescription rate by 13%. Only the IR of OMA episodes remained significantly lower (22%) during the post-restrictions period. The provision of low-value care also changed. The IR of imaging for back or knee pain and low-value prescription of antibiotics for OMA both decreased significantly during the COVID-restrictions period (by 21% and 78%), but only the low-value prescription rate of antibiotics for OMA remained significantly lower (by 63%) during the post-restrictions period. The IR of inappropriately repeated opioid prescriptions remained unchanged over all three periods.

**Conclusions:**

This study shows that both the rate of episodes as well as the rate at which low-value care was provided have generally been affected by the COVID-19 restrictions. Furthermore, it shows that the magnitude of the impact of the restrictions varies depending on the type of low-value care.

This indicates that deimplementation of low-value care requires tailored (multiple) interventions and may not be achieved through a single disruption or intervention alone.

**Supplementary Information:**

The online version contains supplementary material available at 10.1186/s12875-024-02306-7.

## Introduction

The COVID-19 pandemic has greatly impacted healthcare. Governments introduced several social restrictions, such as lockdowns, to prevent the spread of COVID-19 and to mitigate pressure on healthcare systems [1]. Recent studies have shown that during COVID-19, patients have refrained from visiting their general practitioner (GP) [2–7]. A report from the Dutch National Institute for Public Health and the Environment estimated that during first months of COVID-19 (March—June 2020), the number of GP consultations decreased by approximately 11% compared to the same period in 2019 [8]. This decrease in visits has been linked to medical examinations and treatments being delayed, postponed or completely forgone. Additionally, the decrease in GP visits is only partially accounted for by an increase in telemedicine visits [9–13], indicating some patients did not receive the same care they would have received before the pandemic. However, the actual impact of these restrictions applied during the COVID-19 pandemic remains largely unknown.

The COVID-19 restrictions might have resulted in patients missing (necessary) care during the pandemic. Since the COVID-19 pandemic affected both the number of GP visits and provided care, it is also broadly hypothesized that COVID-19 could also have impacted the provision of low-value care among GPs [14–20]. Low-value care is defined as care which offers no net benefit for the patient and could be associated with harmful outcomes and wasteful spending [21–23]. The COVID-19 pandemic therefore might provide an unique opportunity to study changes in high- and low-value care provision, and where changes might be sustained or stopped. During the COVID-19 pandemic, patients could have been shielded from unnecessary or harmful medicine while they were unable to visit their GP or receive treatment. A process which is also referred to as quaternary prevention, thereby improving the quality of care these patients have received [24, 25]. The provision of low-value care could lead to unnecessary time and costs due to additional prescriptions, laboratory tests, extra consultations and referrals [26].

A study from the US indicated that COVID-19 reduced the amount of low-value care provided [27]. Using claims data Shahzad et al., showed that on average the amount of low-value services decreased by 56.2% during the initial month of the pandemic (April 2020), before rebounding to 83.1% of baseline by January of 2021. Unfortunately, apart from this study, knowledge regarding the impact of COVID-19 on the provision of low-value care is limited. Most studies to date have examined its impact on hospital care, Knowledge regarding its impact on (low-value) healthcare provision among GPs is lacking. We therefore studied the effect of COVID-19 on the provision of three types of low-value GP care derived from the Dutch GP guidelines in a primary care practice research network in the Netherlands, using routinely collected healthcare registration data:

1. Use of imaging in the diagnosis of musculoskeletal complaints related to the back or knee [28–30].
2. Prescription of antibiotics for otitis media acuta (OMA) in children without severe symptoms [31].
3. Prescription of repeat opioid prescriptions, without a prior GP visit [32].

Through quantification of the number and rates of both episodes, as well as episodes receiving low-value treatment, before, during and after the peak of COVID-19 to gain insight into its effect on the provision of (low-value) GP care.

## Methods

### Design and database

We conducted a retrospective cohort study using registration data from the database of the department of primary and community care of the Radboud university medical center. This database contains routinely collected registration data of approximately 40.000 registered patients of the GP network FaMe-net (32 GPs, six primary care practices). In FaMe-Net, all morbidity is registered in episodes of care. The title of an episode of care is the episode diagnosis, classified with the ICPC-2. The episode diagnosis can be modified during the episode of care. For example, when abdominal pain turns out to be a colon carcinoma on further diagnosis. Medication prescriptions are recorded using Anatomical Therapeutic Chemical (ATC) codes, and are linked to the relevant episode diagnosis [33]. Data collected between the 1st of January of 2017 and 31st of December 2022 were used to examine the impact of COVID-19 on both the number and rates of episodes and the provision of low-value services for three types of low-value care.

### Outcome measures

The following outcome measures were used to quantify the impact of the COVID-19 pandemic on the occurrence of episodes and the provision of low-value GP care.

1. The rate of episodes or prescriptions recorded during the pre-, COVID-19 and post-restrictions period.
2. The rate of episodes or prescriptions that could be considered of low-value during the pre-, COVID-19 and post-restrictions period.

Incidence rates (IR) were calculated by dividing the total number of (low-value) episodes or prescriptions recorded by the total amount of years patients were present over each period. Thereby correcting for the time patients were able to visit the GP practice. However, apart from calculating the rates for each of these, we also first report on the raw numbers of episodes and low-value care provision recorded as supporting information.

### Selection of the types of low-value GP care & cohort selection

In a previous study, a prioritization was made of "do-not-do" recommendations present in Dutch GP guidelines resulting in a top 30 of recommendations perceived as being highly relevant (through means of an online survey among 5000 GPs) [26]. The resulting top 30 was presented to two authors (ToH and HS), whom are also active as GPs in clinical practice. They selected "do-not-do" recommendations based on their perceived relevance and occurrence in current daily practice. After having discussed the outcomes of their selections, the selected recommendations by both authors were clustered into three topics while multiple recommendations concerned similar topics (see Table 1). Details regarding the operationalization of the topics (e.g. the data definitions) can be found in Additional file 1.

**Table 1:** Short descriptions of the operationalization of the different types of low-value GP care, including from which guidelines they were derived (additional file 1 contains an elaborate description of the specific diagnose codes included):

*1. The use of imaging in the diagnosis of musculoskeletal complaints related to the back or knee*. Dutch GP guidelines do not recommend to order imaging in case of non-specific knee or back pain. For our assessment we selected all episodes related to back or knee pain were selected. Next, all contacts with a code indicating they resulted in an imaging procedure were matched to each episode based on their unique episode identifier. The episodes with an associated contact indicating the performance of an imaging procedure were considered to have received low-value imaging [28–30]
2. *The prescription of antibiotics for otitis media acuta (OMA) in children without severe symptoms*. Guidelines recommend not to prescribe antibiotics in case of otitis media in children without the patient being seriously ill or without them being at risk of complications. For our assessment, we selected all episodes of otitis media acuta among children (< 18 years old). Next, all prescriptions of antimicrobial agents were matched to the distinct OMA episode based on episode number and prescription date. The Dutch GP guidelines only advice the prescription of an antibiotic in case of OMA when no improvement of both the present fever or pain occurs after three days of appropriate pain management. We therefore defined severe symptoms as children which had a reason for encounter for OMA of at least 72 h. In case a child did not have a reason for encounter of at least 72 h, but had received a prescription for antibiotics within this time frame we marked that prescription as being of low-value [31].
3. *Repeat opioid prescriptions, without a prior GP visit*. Guidelines advice repeat opioid prescriptions only to be prescribed following a consultation with a GP. We therefore included all opioid prescriptions over the examined period in our examination of low-value repeated opioid prescriptions in our assessment. In our analysis of appropriateness, we did not include the initial opioid prescriptions, while these simply cannot be considered repeat prescriptions. Next, the identified GP contacts were matched to each of the repeat opioid prescriptions based on their respective contact and prescription dates. These had to match in order for the repeat opioid prescription to be considered as being appropriate. Repeat prescriptions that did not have a contact associated to them were considered as being of low-value [32].

Following operationalization of the different topics, all patients matching the diagnose codes included in our data definitions over the examined period (2017 – 2022) were extracted from the database. We did not limit ourselves to patients visiting the included practices with COVID-19 related complaints. All relevant contact were included for either of the included practices, especially since research has shown that during COVID-19 GP the way GPs were visited substantially changed. With more and more visits being conducted remotely, and less in a face-to-face manner [34, 35]. Furthermore, while within the Dutch healthcare system, all citizens are required by law to be registered at a GP and to have healthcare insurance (covering the costs of GP visits). Furthermore, patients can only gain access to (non-emergency) hospital care through referral of their GP. Guaranteeing that almost all relevant episodes of patients registered at the included practices (apart for medical emergencies) were included in the different cohorts of this study.

### Defining the assessment numerator and denominator: Assessment lenses

Two types of assessment lenses were used depending on the type of care examined: the patient-indication and service lens [36]. The patient-indication lens was applied in our assessment of the inappropriate use of imaging for musculoskeletal problems, and antibiotic prescriptions in case of OMA. Which implies that only patients with a certain indication were included in the denominator for these assessments, the numerator consisted of patients that received the types of low-value care for at least one episode. For our assessment of inappropriate repeat opioid prescriptions a service lens was used. Implying that all registered opioid prescriptions were included in the denominator, and all prescriptions considered to be inappropriately repeated in the numerator.

### Definitions of the Pre-, COVID-19 and Post-restrictions periods

**Fig. 1:**
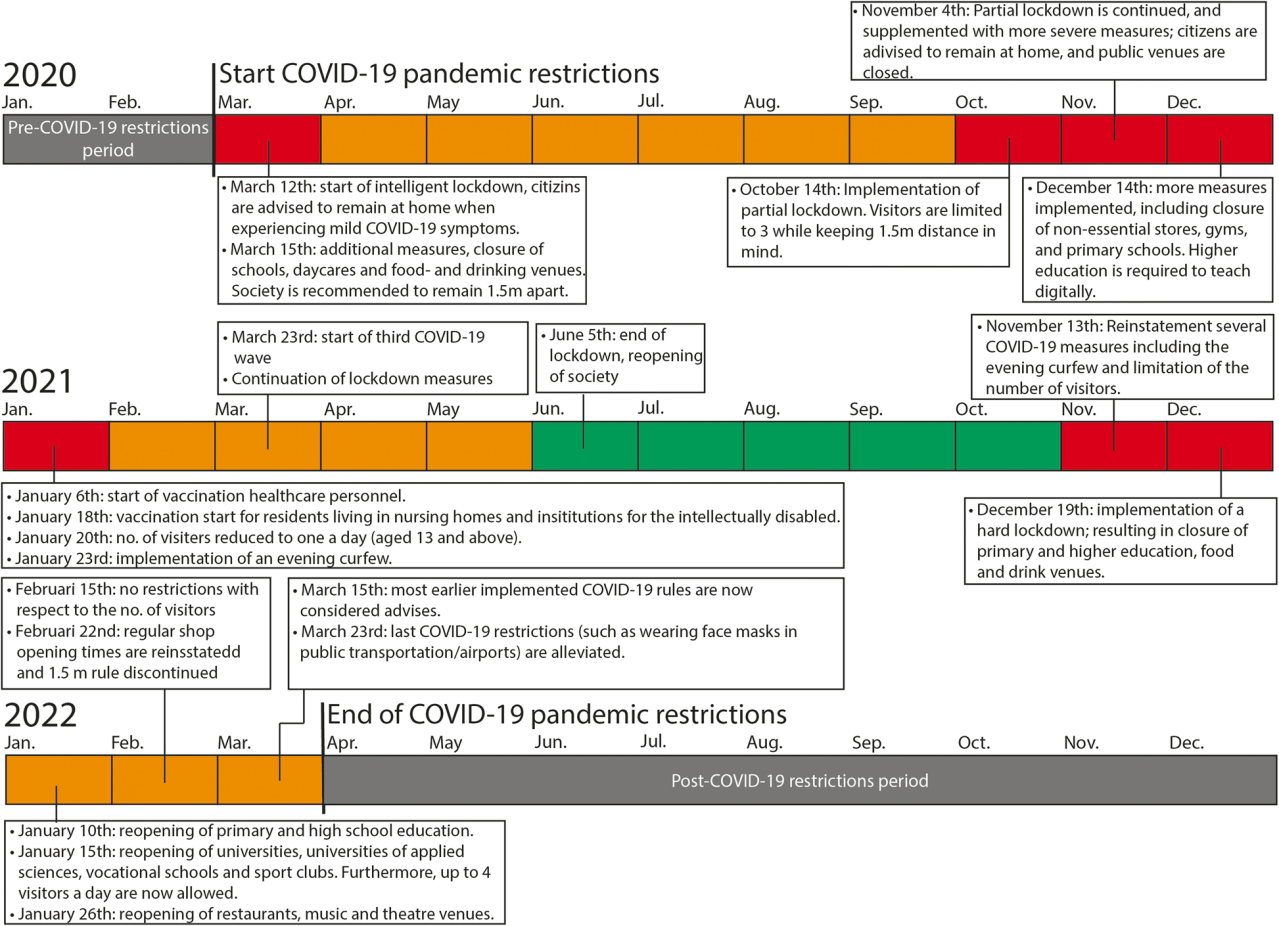
Overview of the timeline and restrictions implemented over the course of the COVID-19 pandemic

We defined the COVID-19 period as the period during which strong COVID-19 related restrictions, such as lockdowns, were imposed on the Dutch population as described on the website of the Dutch Government [37]. Resulting in the period between the 1st of April 2020 (the second quarter of 2020) and the 31st of December 2021 (the fourth quarter of 2021) to be referred to as the COVID-19 period. While the periods before (January 1st 2017 to April 2020) and after the COVID-19 period (the 1st of January 2022 up to the 31st of December 2022) are referred to as the pre- and post-restrictions periods. Figure 1 provides an overview of the timeline and restrictions. Additional file 2 presents a detailed overview of the restrictions used to define the COVID-19 period.

### Data analysis

#### Assessing differences in incidence rates of episodes and low-value care provision

To test the differences in IRs between each of the periods, Poisson multilevel regression models were built, and checked for overdispersion. In case overdispersion was detected, negative binomial models were built to account for the over-dispersed data. Separate models were built to examine whether changes in the IR of episodes/prescriptions or the provision of low-value care could be detected between the three periods. In each of the models a fixed effects of period was included and we aimed to include random effects for both the patient and practice level when possible. Furthermore, we included an offset for the number of years a patient was present in each period, to correct for any differences in duration patients were present over the different periods [38]. Patient age and sex were included as case-mix variables in the models, while previous research has shown they could affect the amount of care a patient requires, receives or has access to [39–41]. Differences in IRs between periods were expressed as Rate Ratio (including 95% confidence intervals [95% CI]). The pre-COVID-19 period was taken as reference period. A P-value smaller or equal to 0.05 was considered statistically significant for all analyses, based on two-sided testing. Data analysis and visualization was performed using R (version 4.1.3).

## Results

### Trends in number of recorded episodes and recorded episodes receiving low-value care

Over the COVID-19 restrictions period, both the number of recorded episodes or prescriptions across the three types of GP care examined show distinct patterns. Table 2 contains an overview of the population characteristics of the populations used to examine each type of care. The average number of episodes and number of episodes receiving low-value care over the periods examined is shown in Table 3 (additional file 3 contains an extended version of Table 3). Both the number of episodes of back and knee pain and prescriptions of antibiotics for OMA sharply decrease at the onset of the COVID-19 period (plots and data concerning separate back and knee episodes are shown in additional file 4). With the average number of episodes of back and knee pain decreasing from 848.4 to 692.9, and episodes of OMA from 145.7 to 99.0.The number of opioid prescriptions also showed to slightly decrease from 988.3 to 1016.8, but already showed to slightly decrease before onset of the restrictions (as shown in additional file 3). The number of episodes of all three types of care show to gradually increase again over the course of the COVID-19 restrictions period.

**Table 2:** Overview of the population characteristics for the different types of care examined

	**Pre-COVID-19 restrictions period**	**COVID-19 restrictions period**	**Post-COVID-19 restrictions period**
**1. The use of imaging in the diagnosis of musculoskeletal complaints related to the back or knee.**			
*Total no. of unique patients, n*	10,802	10,329	9,798
*Female, n (%)*	5,876 (54.4)	5,609 (54.3)	5,271 (53.8)
*Age category, n (%)*			
*0 - 18*	1,057 (9.8)	1,049 (10.2)	1,023 (10.4)
*19 - 50*	4,784 (44.3)	4,729 (45.8)	4,477 (45.7)
*50 - 70*	3,387 (31.4)	3,193 (30.9)	3,078 (31.4)
*70+*	1,574 (14.6)	1,358 (13.1)	1,220 (12.5)
**2. The prescription of antibiotics for otitis media acuta (OMA) in children without severe symptoms.**			
*Total no. of unique patients, n*	1,684	1,875	1,823
*Female, n (%)*	807 (47.9)	881 (47.0)	859 (47.1)
*Age category, n (%) *			
*0 - 1*	637 (37.8)	843 (45.0)	815 (44.7)
*1 - 5*	690 (41.0)	683 (36.4)	669 (36.7)
*5 - 12*	275 (16.3)	271 (14.5)	264 (14.5)
*12 - 18*	82 (4.9)	78 (4.2)	75 (4.1)
**3. Repeat opioid prescriptions. without a prior visit**			
*Total no. of unique patients, n*	2,192	1,285	954
*Female, n (%)*	1,305 (59.5)	778 (60.5)	596 (62.5)
*Age category, n (%) *			
*0 - 50*	430 (33.0)	254 (32.6)	222 (37.2)
*50 - 70*	392 (33.4)	197 (36.6)	136 (35.2)
*70+*	436 (33.6)	285 (30.7)	210 (27.5)

**Table 3:** Average number of episodes and number and proportion of episodes receiving low-value care for the three types of GP care examined

	**Pre-COVID-19 restrictions** **period**	**COVID-19 restrictions** **period**	**Post-COVID-19 restrictions** **period**
**1. The use of imaging in the diagnosis of musculoskeletal complaints related to the back or knee**			
*Average no. of episodes*	848.4	692.9	713.5
*Average no. episodes receiving low-value care*	80.5	62.6	74.0
*Average % of episodes receiving low-value treatment*	9.5	9.0	10.4
**2. The prescription of antibiotics for otitis media acuta (OMA) in children without severe symptoms**			
*Average no. of episodes*	145.7	99.0	182.3
*Average no. episodes receiving low-value care*	9.6	3.0	5.8
*Average % of episodes receiving low-value treatment*	6.8	4.1	3.4
**3. Repeat opioid prescriptions. without a prior visit**			
*Average no. of episodes*	988.3	867.6	1016.8
*Average no. episodes receiving low-value care*	249.2	217.3	244.0
*Average % of episodes receiving low-value treatment*	25.4	25.1	24.0

Regarding low-value treatment (Table 3), both the number of (low-value) imaging procedures and antibiotic prescriptions for OMA slightly decreased (from 80.5 to 62.6, and from 9.6 to 3.0 respectively. In both cases the observed decrease was reverted during the post-restrictions period. The low-value prescription of opioids was not affected by the introduction and removal of the restrictions since its low-value prescription remained high over the entire period remaining relatively high (with an average decrease from 249.2 to 217.3). However, since these raw numbers are not corrected for either exposure period nor any patient characteristics, we have performed our main analysis using the rates of provision as described below.

### Trends in rates of episodes between the different periods

**Fig. 2:**
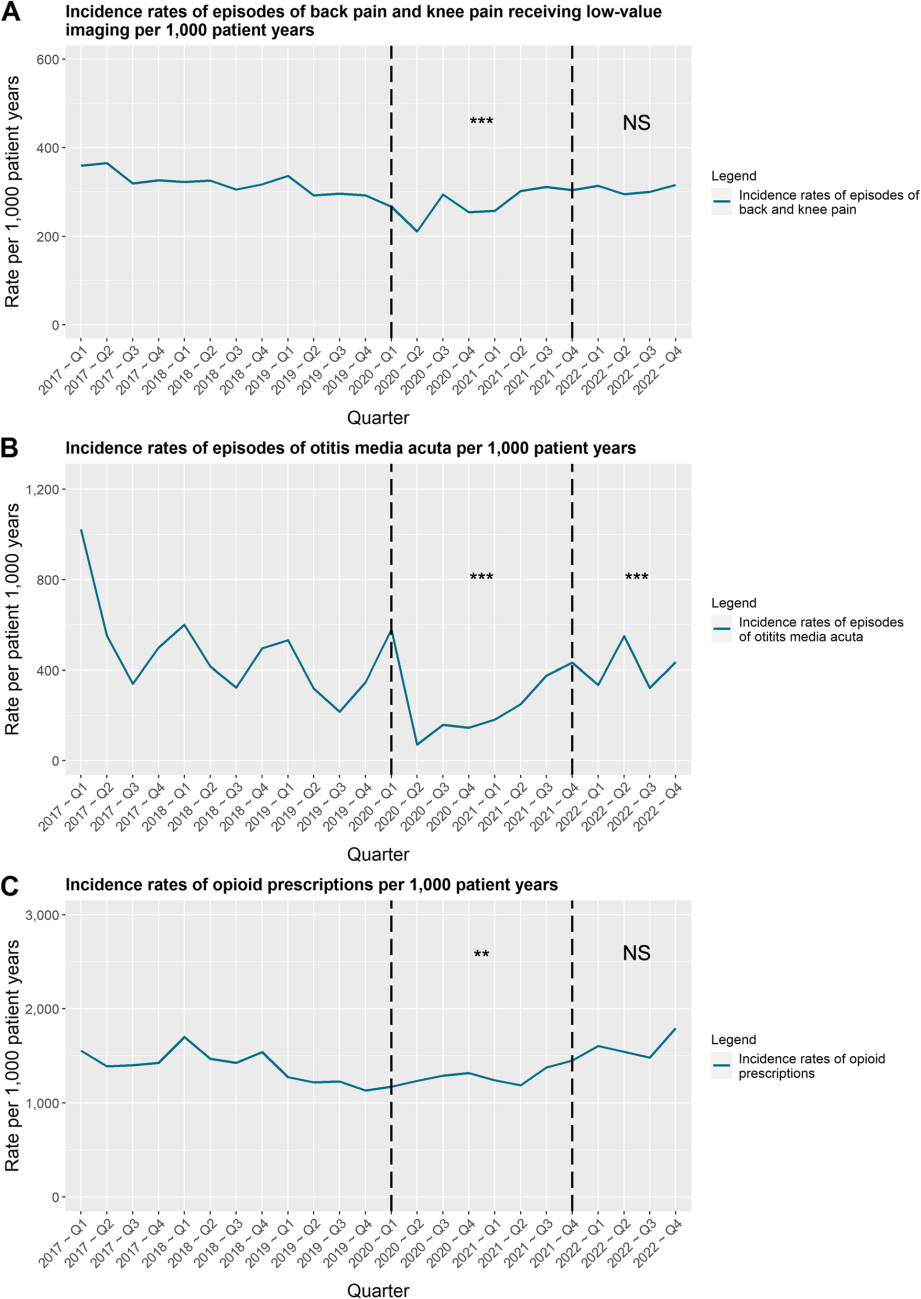
Trends in incidence rates of episodes or prescriptions per 1,000 patient years corresponding to each of the three types care examined. Significance levels: * indicates significance at 0.05 level,** indicates significance at 0.01 level, *** indicates significance at 0.001 level, NS indicates no significance difference was found

The IR of the episodes of the examined types of GP care all significantly decreased over the COVID-19 period (Fig. 2, and Table 4). Both the IRs of back and knee pain and opioid prescriptions only moderately decreased by 12% (*p* < 0.001) and 13% (*p* < 0.01) over the restrictions period. However, these decreases did not sustain during the post-restrictions period. Both the IRs of episodes of back and knee pain and opioid prescriptions did not significantly differ from the pre-restrictions period (*p* > 0.05). In case of OMA among children the IR of episodes decreased by 54% during the restrictions period (*p* < 0.001). In contrast to the other two types of care, the IR of OMA episodes remained significantly (22%) lower during the post-restrictions period (*p* < 0.001). The IR of OMA episodes shows a clear seasonal tendency. The rate of episodes peaks around the first quarter of the included years, apart from the first quarter of 2021.

**Table 4:** Rate ratios over the different periods

	**Rate ratio of episodes /prescriptions (incl. 95% CI)**			**Rate ratio of low-value episodes /prescriptions (incl. 95% CI)**		
	**Pre COVID-19 restrictions period (reference period)**	**COVID-19 restrictions period**	**Post COVID-19 restrictions period**	**Pre COVID-19 restrictions period (reference period)**	**COVID-19 restrictions period**	**Post COVID-19 restrictions period**
*The use of imaging in the diagnosis of musculoskeletal complaints related to the back or knee*	1.0	0.88 *** [0.85 – 0.91]	0.96 [0.92 – 1.0]	1.0	0.79 *** [0.71—0.88]	0.93 [0.83—1.06]
*The prescription of antibiotics for otitis media acuta (OMA) in children without severe symptoms*	1.0	0.46 *** [0.42 – 0.51]	0.78 *** [0.71 – 0.86]	1.0	0.22 *** [0.14 – 0.36]	0.37 *** [0.23 – 0.61]
*Repeat opioid prescriptions, without a prior GP visit*	1.0	0.87 ** [0.79 – 0.96]	1.06 [0.96 – 1.18]	1.0	0.93 [0.79 – 1.09]	1.05 [0.88 – 1.24]

^*^ Significant at 0.05 level,** significant at 0.01 level, *** significant at 0.001 level

### Trends in incidence rates of low-value care between the different periods

**Fig. 3:**
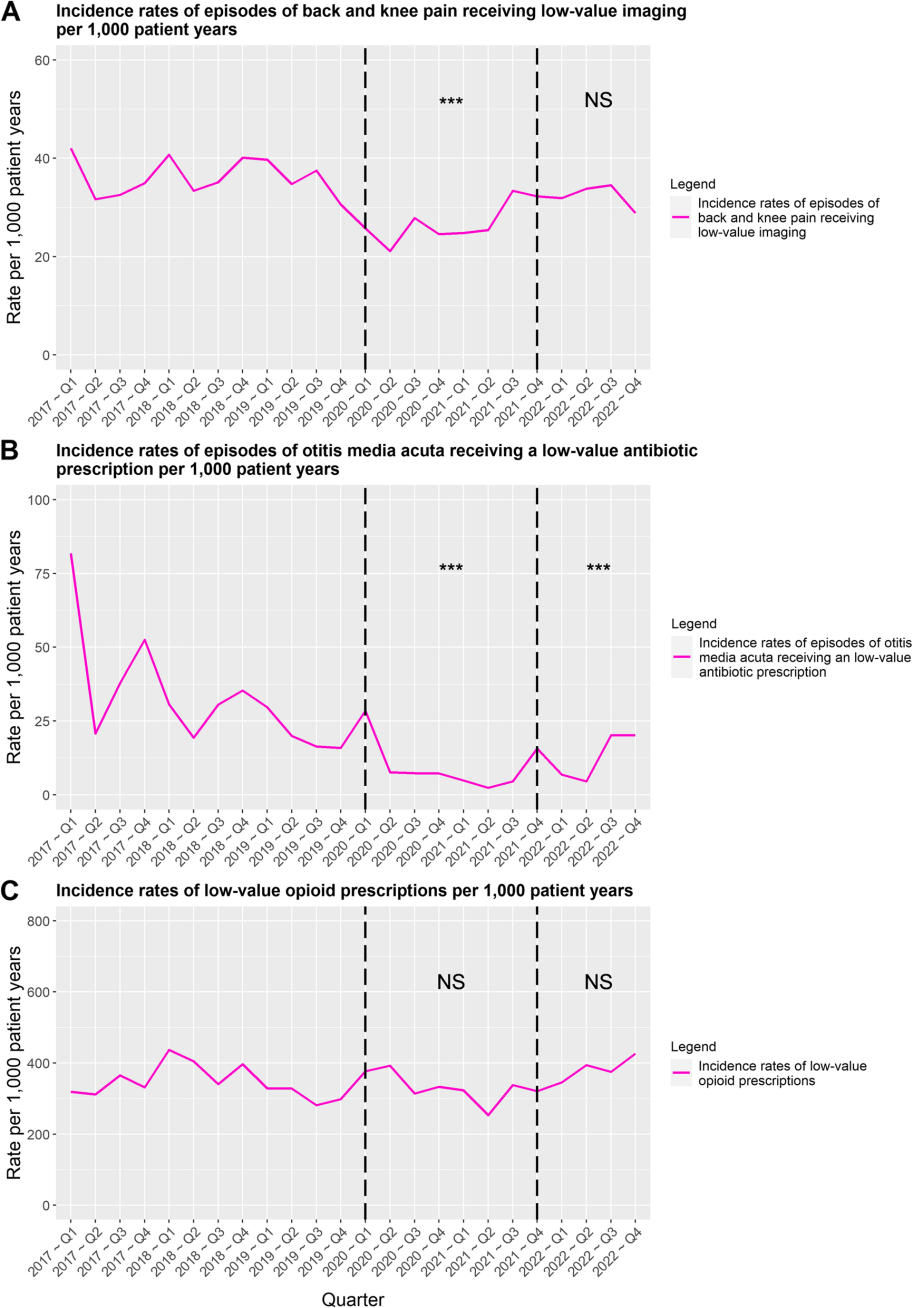
Trends in incidence rates of low-value care provision per 1,000 patient years for the three types of care examined. Significance levels: * indicates significance at 0.05 level,** indicates significance at 0.01 level, *** indicates significance at 0.001 level, NS indicates no significant difference was observed

The IRs of two out of the three types of low-value GP care significantly decreased during the COVID-19 restrictions period (Fig. 3 and Table 4). The IR of episodes of back or knee receiving low-value imaging decreased by 21% (*p* < 0.001) and the IR of OMA episodes receiving low-value antibiotics by 78% (*p* < 0.001). The IR of low-value repeat opioid prescriptions also showed to have decreased by 7%, however did not significantly differ from the IR of the pre-restrictions period (*p* > 0.05). During the post-restrictions period, the IR of low-value imaging for back and knee pain and low-value repeat opioid prescription both returned to pre-restrictions period levels. Conversely, the IR of low-value antibiotics prescriptions for OMA remained 63% lower during the post-restrictions period (*p* < 0.001). In case of the IR of low-value antibiotic prescriptions for OMA in children we did not observe a clear seasonal trend as was the case with the rate of OMA episodes. Additional file 5 contains the IRs for the different types of care examined.

## Discussion

### Summary

This study shows that the COVID-19 related restrictions have affected both the IRs of episodes and low-value care provision among the clinical scenarios examined. The IRs of episodes of all three types of care significantly decreased during the COVID-19 restrictions period. Only the IR of antibiotic prescriptions for OMA remained significantly lower (22%) over the post-COVID-19 restrictions period. The rates at which low-value care was provided during the COVID-19 period, significantly decreased in case of back and knee imaging (21%) and the prescription of antibiotics for OMA (78%). During the post-restrictions period, only the decrease in IR of the low-value antibiotic prescription for OMA remained lower (*p* < 0.001). The IR of inappropriately repeated opioid prescriptions remained unchanged over all three periods (*p* > 0.05).

### Strengths and limitations

This is the first study to examine the effects of the COVID-19 restrictions on the provision of low-value care among GPs. We used routinely collected, highly detailed and high quality clinical information. GPs part of the FaMe-Net meet regularly to discuss and review the coding system to ensure that the quality and validity of data registration remains high [42]. The availability of such reliable and detailed clinical information enabled us to accurately distinguish the appropriateness of the examined clinical scenarios. However, this study is also prone to some limitations. First, this study was conducted using data collected from only 6 out of approximately 4,874 practices in the Netherlands [43]. However, the patient population of the GP practices included in the network has been shown to be representative for the Netherlands with respect to age, sex and social class [44, 45]. We therefore expect our findings to be generalizable to the entire Dutch population. Second, due to the use of a reasonably small network of practices, we were limited with respect to the depth of our analysis. For example, we observed that we had to few data points of our outcome measure over the included months or even quarters to include an seasonal effect in our models. Third, our assessment of low-value repeat opioid prescriptions could be an underestimate as we limited ourselves to the prescriptions prescribed within the time period examined. Some opioid prescriptions might therefore have been wrongly classified as appropriate, while we did not take into account prescriptions prescribed shortly before the inclusion period. However, we do not expect this to have a large impact on the found results while opioids prescriptions are generally short. Fourth, we were only able to include several patient characteristics in our models, but were not able to correct for GP characteristics (such as age and sex). Lastly, we want to note that indeed the operationalization of recommendations in guidelines is often a challenging task, mainly because we were limited with respect to the applicable Dutch guidelines for GPs and subsequent assumptions that had to be made. For example, in our examination of the prescription of antibiotics for OMA in children we had to rely on the information presented to us in the Dutch GP guidelines. Stating that OMA related complaints generally will resolve themselves within 48 – 72 h, and that after 72 h of complaints the prescription of antibiotics is considered appropriate [31]. We were unable to find any other information regarding the proper definition of severe symptoms in case of OMA, applicable to Dutch GP care. Additionally, some countries issued temporary modified guidelines for GP care during COVID-19, such as in long-term pain management with opioids [46]. However, the relevant GP bodies in the Netherlands have not adjusted their guidelines during COVID-19, resulting in us needing to use the existing guidelines.

### Comparison with existing literature

Our findings regarding the observed differences in both trends in episodes [47–51] and low-value services are in line with previous studies in hospital care [27, 52–54]. Hence, both the number and rates of episodes and low-value care provision were largely affected during the first months of the COVID-19 pandemic. Furthermore, our finding that the pandemic differentially affected the provision of the different types of low-value service, complies with assessments regarding the impact of COVID-19 healthcare from the US, albeit it being conducted in hospitals [27]. Hence Shahzad et al. demonstrated that the pandemic had varying effects on low-value care provision, with some types not rebounding afterward.

### Implications for research and/or practice

The results of our assessment show that the introduction of the COVID-19 restrictions have differentially affected low-value GP care. Reasons for which could be found in the severity of the complaints of the different clinical scenarios examined. In both the case of imaging for back or knee pain or the prescription of OMA, the implemented restrictions did not affect the patients’ complaint status. Hence, the symptoms of a patient with back or knee pain do not diminish after having received an imaging procedure. Additionally, OMA related complaints often resolve themselves over time (e.g. 2–3 days) without the prescription of an antibiotic. In both cases, the patient conditions do not necessarily deteriorates but could potentially even improve. Conversely, in case of the prescriptions of opioids, generally the patient’s condition deteriorates while these are often prescribed for patients suffering from long-term or chronic pain syndromes. This notion could provide an explanation as to why we observed that in case of opioids (almost) no change in prescription rates was observed, while the rates of the other types of care did show to change. Furthermore, the observation that the COVID-19 restrictions differentially affected low-value GP care provision supports the idea that deïmplementation of low-value care requires tailored interventions [55, 56]. A recently published review showed that among the existing studies examining the impact of deïmplementation strategies showed that strategies targeting healthcare providers, patients or organizational context are often more effective [55]. Suggesting that the provision of low-value care is often the result of an interplay of factors existing on multiple levels. For example, although healthcare providers often try to provide the best care possible, implemented systems on the level of the hospital could often hinder them in its provision. However, because the COVID-19 pandemic affected the entire healthcare system and was noticeable across all levels of healthcare provision it might have alleviated some of the barriers which earlier prevented the provision of appropriate care.

Future research should investigate both the (potential) mechanisms underlying the observed changes in the IR of low-value care provision over the COVID-19 period for some of the examined types of care, as well as the GPs’ perspective as to why these changes in the IR of low-value care shows such different patterns. Hence, the IR of some of the examined types of care decreased during COVID-19, but rebounded afterwards in some cases (however, this was not the case in the IRs associated to OMA). Additionally, further examination of patient and physician characteristics associated with either the provision or reception of low-value GP care is warranted. While these insights could also be used to further develop interventions aiming to reduce low-value care. Furthermore, exploring whether similar trends can be observed in the use of high-value GP services could also be valuable.

## Conclusion

This study shows that both the IRs of episodes and low-value care provision among Dutch GPs are affected by the COVID-19 restrictions, although differences between the clinical scenarios were identified Additionally, our findings indicate that only in some cases the COVID-19 restrictions could have had a lasting effect on the provision of low-value care. The combination of these findings confirm the idea that reducing low-value care is a complex challenge; which requires tailormade interventions and which is not easily nor quickly achieved.

## Supplementary Information

**Supplementary Material 1. **

## Data Availability

The data that support the findings of this study are available from the department of primary and community care of the Radboud university medical center but restrictions apply to the availability of these data, which were used under license for the current study, and so are not publicly available. Data are however available from the authors upon reasonable request and with permission of the department of primary and community care of the Radboud university medical center.

## Abbreviations

GP: General Practitioner
IR: Incidence Rates
OMA: Otitis Media Acuta
ATC: Anatomical Therapeutic Chemical
