# Correction: Trends in low-value GP care during the COVID-19 pandemic: a retrospective cohort study

**DOI:** 10.1186/s12875-024-02652-6

**Published:** 2024-11-26

**Authors:** Joris L. J. M. Müskens, Tim C. Olde Hartman, Henk J. Schers, Reinier P. Akkermans, Gert P. Westert, Rudolf B. Kool, Simone A. van Dulmen

**Affiliations:** 1 IQ Health Science Department, Radboud University Medical Center, Nijmegen, The Netherlands; 2 Department of Primary and Community Care at Radboud Institute for Health Sciences, Radboud University Medical Center, Nijmegen, The Netherlands

**Correction: BMC Prim. Care 25, 73 (2024)**

10.1186/s12875-024-02306-7.

Following publication of the original article [1], the authors reported that incorrect population statistics were presented in Table 2 for one of the types of low-value care examined, repeat opioids prescriptions. The total number of unique patients for the pre-COVID restrictions period, COVID-19 restrictions period, and post-COVID-19 restrictions period were mistakenly presented as 3,498 (row 18, columns 2–4). The correct numbers of unique patients are now reported in Table 2 as 2,192, 1,285, and 954 respectively alongside the corrected proportions for female, n (%) and age category, n (%) (rows 18–23, columns 2–4).

The authors would also like to correct one line referring to this Table in the Discussion section under ‘Implications for research and/or practice’ which refers to the number of patients with repeat opioid prescriptions remaining the same over the time period. This text has been corrected from “This notion could provide an explanation as to why we observed that in case of opioids (almost) no change in prescription rates was observed, while the rates of the other types of care did show to change (and the number and distribution of patients remained the same over the period examined)” to “This notion could provide an explanation as to why we observed that in case of opioids (almost) no change in prescription rates was observed, while the rates of the other types of care did show to change.”

None of the subsequent analysis or outcomes described in the article were affected by this error.

See below for the correct section and table.

**Incorrect section**:

***Implications for research and/or practice***

The results of our assessment show that the introduction of the COVID-19 restrictions have differentially affected low-value GP care. Reasons for which could be found in the severity of the complaints of the different clinical scenarios examined. In both the case of imaging for back or knee pain or the prescription of OMA, the implemented restrictions did not affect the patients’ complaint status. Hence, the symptoms of a patient with back or knee pain do not diminish after having received an imaging procedure. Additionally, OMA related complaints often resolve themselves over time (e.g. 2–3 days) without the prescription of an antibiotic. In both cases, the patient conditions do not necessarily deteriorates but could potentially even improve. Conversely, in case of the prescriptions of opioids, generally the patient’s condition deteriorates while these are often prescribed for patients suffering from long-term or chronic pain syndromes. This notion could provide an explanation as to why we observed that in case of opioids (almost) no change in prescription rates was observed, while the rates of the other types of care did show to change (and the number and distribution of patients remained the same over the period examined). Furthermore, the observation that the COVID-19 restrictions differentially affected low-value GP care provision supports the idea that deïmplementation of low-value care requires tailored interventions [55, 56]. A recently published review showed that among the existing studies examining the impact of deïmplementation strategies showed that strategies targeting healthcare providers, patients or organizational context are often more effective [55]. Suggesting that the provision of low-value care is often the result of an interplay of factors existing on multiple levels. For example, although healthcare providers often try to provide the best care possible, implemented systems on the level of the hospital could often hinder them in its provision. However, because the COVID-19 pandemic affected the entire healthcare system and was noticeable across all levels of healthcare provision it might have alleviated some of the barriers which earlier prevented the provision of appropriate care.

**Correct section**:

***Implications for research and/or practice***

The results of our assessment show that the introduction of the COVID-19 restrictions have differentially affected low-value GP care. Reasons for which could be found in the severity of the complaints of the different clinical scenarios examined. In both the case of imaging for back or knee pain or the prescription of OMA, the implemented restrictions did not affect the patients’ complaint status. Hence, the symptoms of a patient with back or knee pain do not diminish after having received an imaging procedure. Additionally, OMA related complaints often resolve themselves over time (e.g. 2–3 days) without the prescription of an antibiotic. In both cases, the patient conditions do not necessarily deteriorates but could potentially even improve. Conversely, in case of the prescriptions of opioids, generally the patient’s condition deteriorates while these are often prescribed for patients suffering from long-term or chronic pain syndromes. This notion could provide an explanation as to why we observed that in case of opioids (almost) no change in prescription rates was observed, while the rates of the other types of care did show to change. Furthermore, the observation that the COVID-19 restrictions differentially affected low-value GP care provision supports the idea that deïmplementation of low-value care requires tailored interventions [55, 56]. A recently published review showed that among the existing studies examining the impact of deïmplementation strategies showed that strategies targeting healthcare providers, patients or organizational context are often more effective [55]. Suggesting that the provision of low-value care is often the result of an interplay of factors existing on multiple levels. For example, although healthcare providers often try to provide the best care possible, implemented systems on the level of the hospital could often hinder them in its provision. However, because the COVID-19 pandemic affected the entire healthcare system and was noticeable across all levels of healthcare provision it might have alleviated some of the barriers which earlier prevented the provision of appropriate care.

**Incorrect table**

**Table 2:** Overview of the population characteristics for the different types of care examined

	Pre-COVID-19 restrictions period	COVID-19 restrictions period	Post-COVID-19 restrictions period
**1. The use of imaging in the diagnosis of musculoskeletal complaints related to the back or knee**			
*Total no. of unique patients*,* n*	10,802	10,329	9,798
* Female*,* n (%)*	5,876 (54.4)	5,609 (54.3)	5,271 (53.8)
*Age category*,* n (%)*			
* 0–18*	1,057 (9.8)	1,049 (10.2)	1,023 (10.4)
* 19–50*	4,784 (44.3)	4,729 (45.8)	4,477 (45.7)
* 50–70*	3,387 (31.4)	3,193 (30.9)	3,078 (31.4)
* 70+*	1,574 (14.6)	1,358 (13.1)	1,220 (12.5)
**2. The prescription of antibiotics for otitis media acuta (OMA) in children without severe symptoms**			
*Total no. of unique patients*,* n*	1,684	1,875	1,823
* Female*,* n (%)*	807 (47.9)	881 (47.0)	859 (47.1)
*Age category*,* n (%)*			
* 0–1*	637 (37.8)	843 (45.0)	815 (44.7)
* 1–5*	690 (41.0)	683 (36.4)	669 (36.7)
* 5–12*	275 (16.3)	271 (14.5)	264 (14.5)
* 12–18*	82 (4.9)	78 (4.2)	75 (4.1)
**3. Repeat opioid prescriptions. without a prior visit**			
*Total no. of unique patients*,* n*	3,498	3,498	3,498
* Female*,* n (%)*	2,081 (59.5)	2,081 (59.5)	2,081 (59.5)
*Age category*,* n (%)*			
* 0–50*	1,161 (33.2)	1,161 (33.2)	1,161 (33.2)
* 50–70*	1,279 (36.6)	1,279 (36.6)	1,279 (36.6)
* 70+*	1,058 (30.2)	1,058 (30.2)	1,058 (30.2)

**Correct Table**

**Table 2:** Overview of the population characteristics for the different types of care examined

	Pre-COVID-19 restrictions period	COVID-19 restrictions period	Post-COVID-19 restrictions period
**1. The use of imaging in the diagnosis of musculoskeletal complaints related to the back or knee**			
*Total no. of unique patients*,* n*	10,802	10,329	9,798
* Female*,* n (%)*	5,876 (54.4)	5,609 (54.3)	5,271 (53.8)
*Age category*,* n (%)*			
* 0–18*	1,057 (9.8)	1,049 (10.2)	1,023 (10.4)
* 19–50*	4,784 (44.3)	4,729 (45.8)	4,477 (45.7)
* 50–70*	3,387 (31.4)	3,193 (30.9)	3,078 (31.4)
* 70+*	1,574 (14.6)	1,358 (13.1)	1,220 (12.5)
**2. The prescription of antibiotics for otitis media acuta (OMA) in children without severe symptoms**			
*Total no. of unique patients*,* n*	1,684	1,875	1,823
* Female*,* n (%)*	807 (47.9)	881 (47.0)	859 (47.1)
*Age category*,* n (%)*			
* 0–1*	637 (37.8)	843 (45.0)	815 (44.7)
* 1–5*	690 (41.0)	683 (36.4)	669 (36.7)
* 5–12*	275 (16.3)	271 (14.5)	264 (14.5)
* 12–18*	82 (4.9)	78 (4.2)	75 (4.1)
**3. Repeat opioid prescriptions. without a prior visit**			
*Total no. of unique patients*,* n*	2,192	1,285	954
* Female*,* n (%)*	1,305 (59.5)	778 (60.5)	596 (62.5)
*Age category*,* n (%)*			
* 0–50*	430 (33.0)	254 (32.6)	222 (37.2)
* 50–70*	392 (33.4)	197 (36.6)	136 (35.2)
* 70+*	436 (33.6)	285 (30.7)	210 (27.5)

The original article [1] has been corrected.

## References

[1] Müskens JLJM, Hartman O, Schers TC. Trends in low-value GP care during the COVID-19 pandemic: a retrospective cohort study. BMC Prim Care. 2024;25:73. 10.1186/s12875-024-02306-7. 38418951 10.1186/s12875-024-02306-7 PMC10900726

