# Supplementary material for: Trends in low-value GP care during the COVID-19 pandemic: a retrospective cohort study
Source: BMC Prim Care. 2024 Feb 28;25:73. doi: 10.1186/s12875-024-02306-7 (PMC10900726; doi:10.1186/s12875-024-02306-7)
Supplement: Supplementary file 1 — Supplementary Material 1. [file 12875_2024_2306_MOESM1_ESM.pdf]

Additional file 1: overview of both the included recommendations in each of the topics examined and their operationalization.

1. **The use of imaging in the diagnosis of musculoskeletal complaints related to the back or knee.**

The following do-not-do recommendations were included in the operationalization of the low-value use of imaging in the diagnosis of musculoskeletal complaints related to the back or knee.

- The use of imaging is not recommended in case of non-specific lower back pain. While no clear correlation exists between deviations of the Vertebral column visible on images and the presence of non-specific lower back pain.
- The ordering of imaging (X-ray, CT- or MRI-scan) in patients with sciatica is not recommended, unless there are clear signs of the sciatica originating from a severe cause.
- The use of imaging (such as x-ray or MRI-scan) to diagnose or exclude the existence of knee arthrosis, Prepatellar bursitis, Iliotibial band syndrome, Patellar Tendinitis/ Jumper's Knee or Osgood–Schlatter disease is not recommended, since the relation between deviations found during imaging procedures and the patient’s complaints remains unclear.

ICPC-2 codes included	ICD-10 codes included	Operationalisation of recommendations
a. Back symptom/complaint (L02)<br>b. Low back symptom/complaints [ex. L86] (L03)<br>c. Back syndrome without radiating pain (L84)<br>d. Acquired deformity of spine (L85)<br>e. Back syndrome with radiating pain (L86)<br>f. Sprain/strain of knee (L78)<br>g. Knee symptom/complaint (L15)<br>h. Bursitis/tendinitis/synovitis not otherwise specified (L87)<br>i. Osteoarthrosis of knee (L90)<br>j. Osgood–Schlatter disease (L94)<br>k. Musculoskeletal disease other (L99)	a. Patellofemoral disorders (M22.2)<br>b. Prepatellar bursitis (M70.4)<br>c. Iliotibial band syndrome (M76.3)<br>d. Osgood–Schlatter disease (M92.5)<br>e. Patellar Tendinitis/ Jumper's Knee (M76.50)	The International Classification of Primary Care, 2nd edition (ICPC-2) and International Classification of Diseases and Related Health Problems (ICD-10) codes related to back and knee pain as shown on the left were included in our definition of musculoskeletal complaints.<br><br>All episodes which had either of the described ICD-10 or ICPC-2 codes associated to them were extracted from the database and included in our analysis. Next, all contacts having a code indicating they resulted in a imaging procedure associated to them were matched to each episode based on the unique episode identifier. After which we marked all episodes that had such a contact associated to them, as having received imaging. And these therefore were considered as having received low-value imaging.<br><br>The following age categories were used in our examination of this type of low-value use of imaging in case of musculoskeletal problems: 0-18, 18-50, 50-70 and 70+.

2. **The prescription of antibiotics for otitis media acuta (OMA) in children without severe symptoms.**<br>The following do-not-do recommendations were included in the operationalization of the low-value prescription of antibiotics for otitis media acuta in children.<br>• In children with an episode of otitis media without major general symptoms of illness (fever) and without any risk of complications, treatment with an oral antimicrobial agent is not indicated.		
**ICPC-2 codes included**	**ATC codes included**	**Operationalisation of recommendations**
a. Otitis Media Acuta / Myringitis (H71)	a. All ATC codes belonging to J01 (Antibacterials for systemic use):<br>- J01A Tetracyclines<br>- J01B Amphenicols<br>- J01C Beta-lactam antibacterials, penicillins<br>- J01D Other Beta-lactam antibacterials<br>- J01E Sulfonamides and trimethoprim<br>- J01F Macrolides, lincosamides and streptogramins<br>- J01G Aminoglycoside antibacterials<br>- J01M Quinolone antibacterials<br>- J01R Combinations of antibacterials<br>- J01X Other antibacterials	First, all children (age < 18) with an episodes of otitis media acuta (OMA) were extracted from the database. Next, all prescriptions of antimicrobial agents were matched to the distinct OMA episode based on episode number and prescription date. The Dutch GP guidelines only advice the prescription of an antibiotic in case of OMA when no improvement of both the present fever or pain occurs after three days of appropriate pain management. We therefore defined S severe symptoms were as defined as cchildren which had a reason for encounter for OMA of at least 72 hours. This means that the child has experienced OMA related complaints for at least 72 hours. In case a child did not have a reason for encounter of at least 72 hours, but had but did received a prescription for antibiotics within this time frame, we marked that prescription as being of low-value.<br><br>In our examination of the prescription of antibiotics for otitis media acuta (OMA) in children we only included patients aged 18 or less. This resulted in us using the following age categories in our examination: 0-1, 1-5, 5-12, 12-18.

**3. Repeat opioid prescriptions, without a prior GP visit.**<br>The following do-not-do recommendations were included in the operationalization of repeat opioid prescriptions, without a prior GP visit.<br>• Do not automatically repeat opioids, without a prior GP consult. Ask for pain mitigation and stop with prescribing opioids when the pain mitigation is considered to be inadequate.<br>• Try to limit the time an opioid is prescribed to reduce the risk of side effects and excessive use because of habituation and dependency.		
**ICPC-2 codes included**	**ATC codes included**	**Operationalisation of recommendations**
N.A.	a. All ATC codes belonging to N02A (Opioids):<br>- N02AA: Natural opium alkaloids<br>- N02AB: Phenylpiperidine derivatives<br>- N02AC Diphenylpropylamine derivatives<br>- N02AD Benzomorphan derivatives<br>- N02AE Oripavine derivatives<br>- N02AF Morphinan derivatives<br>- N02AG Opioids in combination with antispasmodics<br>- N02AJ Opioids in combination with non-opioid analgesics<br>- N02AX Other opioids	All patients that received an opioid prescription over the examined period were included in our analysis. We did not include the initial opioid prescriptions in our analysis, while these simply cannot be considered a repeat prescriptions.<br><br>In our analysis of the appropriateness of repeat opioid prescriptions, the following GP contacts were included as justifying an repeat prescription: consultations, visits, telephone and email contacts to both GP practices and after hours clinics. Next, the identified GP contacts were matched to their respective repeat opioid prescription based on the contact and prescription dates. These had to match in order for the repeat opioid prescription to be considered as being appropriate. Repeat prescriptions that did not have a contact associated to them were considered as being of low-value.<br><br>In our examination of inappropriate repeat opioid prescriptions, we aimed to apply the same age categories as was the case for the inappropriate imaging assessment. However, there were too little number of prescriptions in the lowest age category. We therefore ended up having to merge the two lowest categories resulting in the following age division: 0-50, 50-70 and 70+.

Additional file 2: overview of COVID-19 restrictions implemented over the examined period

1. **2020:**
   - March 12th: first restrictions implemented to prevent spread of the COVID-19 virus; intelligent lockdown, Dutch citizens are advised to remain at home when experiencing mild COVID-19 symptoms.
   - March 15th: closure of food and drinking venues, schools and day-cares. Society is recommended to keep at least 1,5m distance to one another.
   - October 14th: second wave of COVID-19, partial lockdown is implemented. Facemasks are made mandatory in public domains and transport, and a maximum number of three people that are allowed to be received at home. All food and drinking venues are required to close their business until further notice (take away still a possibility).
   - November 4th: the initial restrictions do not reduce the amount of COVID-19 infections, more severe restrictions are therefore implemented. The Dutch population is advised to remain at home when possible. Furthermore, visitors both inside and outside are further limited to two a day, or one household and public accessible venues are closed. Exercising is limited to two persons (group workouts are prohibited), while remaining at 1.5 m distance (exception for children up to 17 years old and professional athletes)
   - December 14th: Closure of non-essential stores, gyms, day-cares and primary schools. Other schools are required to teach digitally.
2. **2021:**
   - January 6th: start of vaccination campaign among healthcare personnel.
   - January 20rd: Visiting policy tightened to only 1 visitor (aged 13 and older), once a day.
   - January 23rd: evening curfew for the entire Dutch population is implemented, and continuation of the lockdown that started in the previous month. Vaccination of the Dutch population is started.
   - March 23rd: Start third COVID-19 wave, continuation of the lockdown restrictions.
   - June 5th: end of lockdown, most restrictions are revoked and society is re-opened.
   - November 13th: after a sharp increase in COVID-19 related infections, several COVID-19 restrictions are reinstated including an a (partial) evening lockdown (from 05:00 P.M. most non-essential venues are closed). Furthermore, society is advised to work from home again and the number visitors (aged 13 and over) is limited to four per household.
   - December 19th: Hard lockdown is implemented, following detection of the Omicron variant of COVID-19. Resulting in the closure of primary and higher education (high schools, universities and vocational schools), food and drinking venues, non-essential stores, sport club, cultural venues and all public meeting places.
3. **2022:**
   - January 10th: first alleviations of the COVID-19 restrictions implemented during the hard lockdown of 2021 are announced; reopening of both primary and high school education and day-cares.
   - January 15th: reopening of universities, universities of applied sciences and vocational schools and most sport clubs and shops, shops are allowed to open until 5 pm in the afternoon. An general advise is given to wear facemasks when 1,5m distance cannot be uphold. Furthermore, up to 4 visitors are allowed each day, excluding children up until 12 years old.

- January 26th: reopening of restaurants, music and theatre venues (including zoos, cinemas, museums and amusement parks.
- February 15th: no more restrictions with respect to visitors. Furthermore, remote working is no longer mandatory, up to half the time one is now allowed to work at the office.
- February 22nd: Regular opening times for shops are reintroduced and the 1,5m distance rule is discontinued.
- March 15th: COVID-19 rules are now considered to be advises.
- March 23rd: last COVID-19 restrictions (such as mandatory masks required in public transportation) are alleviated.
- April 1st: No more COVID-19 restrictions implemented.

Additional file 3: extended versions showing both the total no. of episodes/prescriptions and low-value episodes/prescriptions as a whole and separated for each of the types of care examined.

1. *Total number of episodes and number and proportion of episodes receiving low-value care for the three types of GP care examined.*

	Pre-COVID-19 restrictions period													COVID-19 restrictions period							Post-COVID-19 restrictions period			
**Year and quarter**	2017 Q1	2017 Q2	2017 Q3	2017 Q4	2018 Q1	2018 Q2	2018 Q3	2018 Q4	2019 Q1	2019 Q2	2019 Q3	2019 Q4	2020 Q1	2020 Q2	2020 Q3	2020 Q4	2021 Q1	2021 Q2	2021 Q3	2021 Q4	2022 Q1	2022 Q2	2022 Q3	2022 Q4
**1. The use of imaging in the diagnosis of musculoskeletal complaints related to the back or knee.**																								
No. of episodes	955	955	882	894	861	880	823	887	881	777	785	750	699	544	751	651	661	750	764	729	750	688	699	717
No. episodes receiving low-value care	92	69	71	79	92	78	81	98	86	79	89	70	62	49	68	58	58	58	77	70	74	78	78	66
% of episodes receiving low-value treatment	9.6	7.2	8.0	8.8	10.7	8.9	9.8	11.0	9.8	10.2	11.3	9.3	8.9	9.0	9.1	8.9	8.8	7.7	10.1	9.6	9.9	11.3	11.2	9.2
**2. The prescription of antibiotics for otitis media acuta (OMA) in children without severe symptoms.**																								
No. of episodes	225	133	90	143	176	129	106	168	179	112	79	131	223	28	65	61	75	106	165	193	147	244	144	194
No. episodes receiving low-value care	18	5	10	15	9	6	10	12	10	7	6	6	11	3	3	3	2	1	2	7	3	2	9	9
% of episodes receiving low-value treatment	8.0	3.8	11.1	10.5	5.1	4.7	9.4	7.1	5.6	6.3	7.6	4.6	4.9	10.7	4.6	4.9	2.7	0.9	1.2	3.6	2.0	0.8	6.3	4.6
**3. Repeat opioid prescriptions, without a prior visit**																								
No. of episodes	1,096	997	1,023	1,049	1,233	1,080	1,067	1,151	866	839	855	787	805	843	883	893	814	780	908	952	1023	983	939	1,122
No. episodes receiving low-value care	225	224	267	244	317	298	255	296	224	226	196	208	259	268	215	226	212	166	223	211	220	251	238	267
% of episodes receiving low-value treatment	20.5	22.5	26.1	23.3	25.7	27.6	23.9	25.7	25.9	26.9	22.9	26.4	32.2	31.8	24.3	25.3	26.0	21.3	24.6	22.2	21.5	25.5	25.3	23.8

2. <u>Number of (low-value) episodes/prescriptions separated for each of the types of care examined.</u>

A) The use of imaging in the diagnosis of musculoskeletal complaints related to the back or knee.

**Year and quarter**	2017 - Q1		2017 - Q2		2017 - Q3		2017 - Q4		2018 - Q1		2018 - Q2		2018 - Q3		2018 - Q4	
**1. The use of imaging in the diagnosis of musculoskeletal complaints related to the back or knee.**																
No. of episodes - age categoriess	**M**	**F**	**M**	**F**	**M**	**F**	**M**	**F**	**M**	**F**	**M**	**F**	**M**	**F**	**M**	**F**
0-18	22	22	32	27	16	22	29	21	31	23	20	30	22	29	36	29
19-50	181	265	153	248	174	234	196	224	166	237	171	238	151	206	169	224
50-70	136	162	143	192	130	147	120	165	110	160	128	154	117	155	126	160
70+	68	99	77	83	76	83	67	72	47	87	68	71	67	76	55	88
**Total no. of episodes**	407	548	405	550	396	486	412	482	354	507	387	493	357	466	386	501
No. episodes receiving low-value care - age categories	**M**	**F**	**M**	**F**	**M**	**F**	**M**	**F**	**M**	**F**	**M**	**F**	**M**	**F**	**M**	**F**
0-18	3	0	1	2	1	2	2	1	4	2	2	1	2	2	4	4
% low-value	13.64	0.00	3.13	7.41	6.25	9.09	6.90	4.76	12.90	8.70	10.00	3.33	9.09	6.90	11.11	13.79
19-50	11	23	12	7	6	18	8	13	8	16	9	16	8	10	15	20
% low-value	6.08	8.68	7.84	2.82	3.45	7.69	4.08	5.80	4.82	6.75	5.26	6.72	5.30	4.85	8.88	8.93
50-70	9	22	13	15	10	13	15	22	10	27	12	23	21	17	15	19
% low-value	6.62	13.58	9.09	7.81	7.69	8.84	12.50	13.33	9.09	16.88	9.38	14.94	17.95	10.97	11.90	11.88
70+	11	13	6	13	11	10	5	13	6	19	7	8	10	11	9	12
% low-value	16.18	13.13	7.79	15.66	14.47	12.05	7.46	18.06	12.77	21.84	10.29	11.27	14.93	14.47	16.36	13.64
**Total no of episodes with low-value treatment**	34	58	32	37	28	43	30	49	28	64	30	48	41	40	43	55
% low-value	8.35	10.58	7.90	6.73	7.07	8.85	7.28	10.17	7.91	12.62	7.75	9.74	11.48	8.58	11.14	10.98

Year and quarter	2019 - Q1		2019 - Q2		2019 - Q3		2019 - Q4		2020 - Q1		2020 - Q2		2020 - Q3		2020 - Q4	
**1. The use of imaging in the diagnosis of musculoskeletal complaints related to the back or knee.**																
No. of episodes - age categoriess	**M**	**F**	**M**	**F**	**M**	**F**	**M**	**F**	**M**	**F**	**M**	**F**	**M**	**F**	**M**	**F**
0-18	31	41	40	26	30	42	27	37	19	30	16	11	34	25	29	29
19-50	162	241	172	177	147	179	161	211	125	177	95	142	135	191	129	156
50-70	122	157	108	140	114	148	97	119	118	126	76	110	126	114	91	120
70+	58	69	54	60	48	77	40	58	53	51	39	55	45	81	45	52
**Total no. of episodes**	373	508	374	403	339	446	325	425	315	384	226	318	340	411	294	357
No. episodes receiving low-value care - age categories	**M**	**F**	**M**	**F**	**M**	**F**	**M**	**F**	**M**	**F**	**M**	**F**	**M**	**F**	**M**	**F**
0-18	1	3	3	1	2	5	2	3	0	2	3	1	2	0	3	2
% low-value	3.23	7.32	7.50	3.85	6.67	11.90	7.41	8.11	0.00	6.67	18.75	9.09	5.88	0.00	10.34	6.90
19-50	11	17	14	14	11	14	9	18	11	13	9	9	13	13	8	12
% low-value	6.79	7.05	8.14	7.91	7.48	7.82	5.59	8.53	8.80	7.34	9.47	6.34	9.63	6.81	6.20	7.69
50-70	16	18	12	20	17	21	10	15	10	11	9	11	11	14	12	11
% low-value	13.11	11.46	11.11	14.29	14.91	14.19	10.31	12.61	8.47	8.73	11.84	10.00	8.73	12.28	13.19	9.17
70+	8	12	4	11	4	15	6	7	8	7	0	7	3	12	4	6
% low-value	13.79	17.39	7.41	18.33	8.33	19.48	15.00	12.07	15.09	13.73	0.00	12.73	6.67	14.81	8.89	11.54
**Total no of episodes with low-value treatment**	36	50	33	46	34	55	27	43	29	33	21	28	29	39	27	31
% low-value	9.65	9.84	8.82	11.41	10.03	12.33	8.31	10.12	9.21	8.59	9.29	8.81	8.53	9.49	9.18	8.68

**Year and quarter**	2021 - Q1		2021 - Q2		2021 - Q3		2021 - Q4		2022 - Q1		2022 - Q2		2022 - Q3		2022 - Q4	
**1. The use of imaging in the diagnosis of musculoskeletal complaints related to the back or knee.**																
No. of episodes - age categoriess	**M**	**F**	**M**	**F**	**M**	**F**	**M**	**F**	**M**	**F**	**M**	**F**	**M**	**F**	**M**	**F**
0-18	32	30	31	41	33	32	32	30	38	38	46	39	27	39	28	43
19-50	139	155	142	176	165	207	153	194	152	201	144	146	149	172	131	187
50-70	101	110	97	147	102	120	104	116	100	119	100	112	105	120	101	129
70+	38	56	51	65	51	54	51	49	43	59	35	66	35	52	40	58
**Total no. of episodes**	310	351	321	429	351	413	340	389	333	417	325	363	316	383	300	417
No. episodes receiving low-value care - age categories	**M**	**F**	**M**	**F**	**M**	**F**	**M**	**F**	**M**	**F**	**M**	**F**	**M**	**F**	**M**	**F**
0-18	2	4	2	5	0	2	2	4	5	1	4	3	2	4	1	4
% low-value	6.25	13.33	6.45	12.20	0.00	6.25	6.25	13.33	13.16	2.63	8.70	7.69	7.41	10.26	3.57	9.30
19-50	9	13	9	9	11	18	7	15	12	19	10	8	12	24	14	17
% low-value	6.47	8.39	6.34	5.11	6.67	8.70	4.58	7.73	7.89	9.45	6.94	5.48	8.05	13.95	10.69	9.09
50-70	15	5	7	12	16	19	7	18	7	19	13	24	12	16	6	12
% low-value	14.85	4.55	7.22	8.16	15.69	15.83	6.73	15.52	7.00	15.97	13.00	21.43	11.43	13.33	5.94	9.30
70+	5	5	6	8	2	9	10	7	5	6	6	10	4	4	4	8
% low-value	13.16	8.93	11.76	12.31	3.92	16.67	19.61	14.29	11.63	10.17	17.14	15.15	11.43	7.69	10.00	13.79
**Total no of episodes with low-value treatment**	31	27	24	34	29	48	26	44	29	45	33	45	30	48	25	41
% low-value	10.00	7.69	7.48	7.93	8.26	11.62	7.65	11.31	8.71	10.79	10.15	12.40	9.49	12.53	8.33	9.83

B) The prescription of antibiotics for otitis media acuta (OMA) in children without severe symptoms.

**Year and quarter**	2017 - Q1		2017 - Q2		2017 - Q3		2017 - Q4		2018 - Q1		2018 - Q2		2018 - Q3		2018 - Q4	
**2. The prescription of antibiotics for otitis media acuta (OMA) in children without severe symptoms.**																
No. of episodes - age categories	**M**	**F**	**M**	**F**	**M**	**F**	**M**	**F**	**M**	**F**	**M**	**F**	**M**	**F**	**M**	**F**
0-1	50	27	30	22	22	12	44	26	39	41	45	24	25	22	36	49
1-5	51	48	25	28	8	19	30	27	27	40	26	19	12	11	31	28
5-12	28	16	9	12	11	14	5	7	10	17	5	5	16	11	9	7
12-18	1	4	5	2	1	3	0	4	2	0	2	3	2	7	3	5
**Total no. of episodes**	130	95	69	64	42	48	79	64	78	98	78	51	55	51	79	89
No. episodes receiving low-value care - age categories	**M**	**F**	**M**	**F**	**M**	**F**	**M**	**F**	**M**	**F**	**M**	**F**	**M**	**F**	**M**	**F**
0-1	7	2	0	2	2	0	3	0	2	1	2	0	0	2	4	3
% low-value	14.00	7.41	0.00	9.09	9.09	0.00	6.82	0.00	5.13	2.44	4.44	0.00	0.00	9.09	11.11	6.12
1-5	4	2	1	0	0	3	9	2	3	1	1	1	0	2	2	2
% low-value	7.84	4.17	4.00	0.00	0.00	15.79	30.00	7.41	11.11	2.50	3.85	5.26	0.00	18.18	6.45	7.14
5-12	3	0	0	0	4	1	0	1	1	0	0	2	2	3	0	0
% low-value	10.71	0.00	0.00	0.00	36.36	7.14	0.00	14.29	10.00	0.00	0.00	40.00	12.50	27.27	0.00	0.00
12-18	0	0	0	2	0	0	0	0	1	0	0	0	0	1	0	1
% low-value	0.00	0.00	0.00	100.00	0.00	0.00	0.00	0.00	50.00	0.00	0.00	0.00	0.00	14.29	0.00	20.00
Total no of episodes with low-value treatment	14	4	1	4	6	4	12	3	7	2	3	3	2	8	6	6
% low-value	10.77	4.21	1.45	6.25	14.29	8.33	15.19	4.69	8.97	2.04	3.85	5.88	3.64	15.69	7.59	6.74

Year and quarter	2019 - Q1		2019 - Q2		2019 - Q3		2019 - Q4		2020 - Q1		2020 - Q2		2020 - Q3		2020 - Q4	
**2. The prescription of antibiotics for otitis media acuta (OMA) in children without severe symptoms.**																
No. of episodes - age categories	**M**	**F**	**M**	**F**	**M**	**F**	**M**	**F**	**M**	**F**	**M**	**F**	**M**	**F**	**M**	**F**
0-1	45	44	26	25	16	19	38	38	51	50	8	9	18	13	25	14
1-5	40	32	22	13	9	11	17	21	51	49	4	1	9	14	11	9
5-12	6	11	8	8	9	9	6	6	1	17	3	3	3	4	1	0
12-18	0	1	8	2	3	3	0	5	2	2	0	0	1	3	0	1
**Total no. of episodes**	91	88	64	48	37	42	61	70	105	118	15	13	31	34	37	24
No. episodes receiving low-value care - age categories	**M**	**F**	**M**	**F**	**M**	**F**	**M**	**F**	**M**	**F**	**M**	**F**	**M**	**F**	**M**	**F**
0-1	4	1	0	2	1	3	1	1	4	1	0	0	0	1	0	2
% low-value	8.89	2.27	0.00	8.00	6.25	15.79	2.63	2.63	7.84	2.00	0.00	0.00	0.00	7.69	0.00	14.29
1-5	4	1	1	0	0	1	2	0	2	2	1	0	0	0	0	0
% low-value	10.00	3.13	4.55	0.00	0.00	9.09	11.76	0.00	3.92	4.08	25.00	0.00	0.00	0.00	0.00	0.00
5-12	0	0	3	0	0	1	0	2	1	1	0	2	2	0	0	0
% low-value	0.00	0.00	37.50	0.00	0.00	11.11	0.00	33.33	100.00	5.88	0.00	66.67	66.67	0.00	0.00	0.00
12-18	0	0	1	0	0	0	0	0	0	0	0	0	0	0	0	1
% low-value	0.00	0.00	12.50	0.00	0.00	0.00	0.00	0.00	0.00	0.00	0.00	0.00	0.00	0.00	0.00	0.00
Total no of episodes with low-value treatment	8	2	5	2	1	5	3	3	7	4	1	2	2	1	0	3
% low-value	8.79	2.27	7.81	4.17	2.70	11.90	4.92	4.29	6.67	3.39	6.67	15.38	6.45	2.94	0.00	12.50

Year and quarter	2021 - Q1		2021 - Q2		2021 - Q3		2021 - Q4		2022 - Q1		2022 - Q2		2022 - Q3		2022 - Q4	
**2. The prescription of antibiotics for otitis media acuta (OMA) in children without severe symptoms.**																
No. of episodes - age categories	**M**	**F**	**M**	**F**	**M**	**F**	**M**	**F**	**M**	**F**	**M**	**F**	**M**	**F**	**M**	**F**
0-1	23	25	46	22	57	35	77	39	43	21	67	46	33	19	28	55
1-5	7	16	18	13	34	20	31	22	43	25	42	42	36	21	52	39
5-12	1	3	5	2	6	4	3	10	4	10	18	17	23	8	7	9
12-18	0	0	0	0	2	7	4	7	1	0	5	7	1	3	3	1
**Total no. of episodes**	31	44	69	37	99	66	115	78	91	56	132	112	93	51	90	104
No. episodes receiving low-value care - age categories	**M**	**F**	**M**	**F**	**M**	**F**	**M**	**F**	**M**	**F**	**M**	**F**	**M**	**F**	**M**	**F**
0-1	0	0	1	0	0	0	1	2	2	0	1	1	1	1	0	3
% low-value	0.00	0.00	2.17	0.00	0.00	0.00	1.30	5.13	4.65	0.00	1.49	2.17	3.03	5.26	0.00	5.45
1-5	0	1	0	0	0	0	2	0	0	1	0	0	2	2	3	3
% low-value	0.00	6.25	0.00	0.00	0.00	0.00	6.45	0.00	0.00	4.00	0.00	0.00	5.56	9.52	5.77	7.69
5-12	0	1	0	0	1	0	0	1	0	0	0	0	2	0	0	0
% low-value	0.00	33.33	0.00	0.00	16.67	0.00	0.00	10.00	0.00	0.00	0.00	0.00	8.70	0.00	0.00	0.00
12-18	0	0	0	0	0	1	1	0	0	0	0	0	0	1	0	0
% low-value	0.00	0.00	0.00	0.00	0.00	14.29	25.00	0.00	0.00	0.00	0.00	0.00	0.00	0.00	0.00	0.00
Total no of episodes with low-value treatment	0	2	1	0	1	1	4	3	2	1	1	1	5	4	3	6
% low-value	0.00	4.55	1.45	0.00	1.01	1.52	3.48	3.85	2.20	1.79	0.76	0.89	5.38	7.84	3.33	5.77

C) Repeat opioid prescriptions, without a prior visit.

**Year and quarter**	2017 - Q1		2017 - Q2		2017 - Q3		2017 - Q4		2018 - Q1		2018 - Q2		2018 - Q3		2018 - Q4	
**3. Repeat opioid prescriptions, without a prior visit**																
No. of episodes - age categories	**M**	**F**	**M**	**F**	**M**	**F**	**M**	**F**	**M**	**F**	**M**	**F**	**M**	**F**	**M**	**F**
0-50	68	133	57	118	73	119	69	93	80	146	69	173	75	174	116	214
50-70	217	176	182	183	167	182	154	198	174	242	163	209	158	193	175	189
70+	188	314	175	282	190	292	174	361	194	397	150	316	146	321	168	289
**Total no. of episodes**	473	623	414	583	430	593	397	652	448	785	382	698	379	688	459	692
No. episodes receiving low-value care - age categories	**M**	**F**	**M**	**F**	**M**	**F**	**M**	**F**	**M**	**F**	**M**	**F**	**M**	**F**	**M**	**F**
0-50	10	22	10	26	21	25	26	14	14	24	20	45	24	37	50	52
% low-value	14.71	16.54	17.54	22.03	28.77	21.01	37.68	15.05	17.50	16.44	28.99	26.01	32.00	21.26	43.10	24.30
50-70	51	34	53	28	51	48	34	42	44	61	44	75	46	41	46	37
% low-value	23.50	19.32	29.12	15.30	30.54	26.37	22.08	21.21	25.29	25.21	26.99	35.89	29.11	21.24	26.29	19.58
70+	45	63	47	60	49	73	44	84	61	113	28	86	44	63	49	62
% low-value	23.94	20.06	26.86	21.28	25.79	25.00	25.29	23.27	31.44	28.46	18.67	27.22	30.14	19.63	29.17	21.45
Total no of episodes with low-value treatment	106	119	110	114	121	146	104	140	119	198	92	206	114	141	145	151
% low-value	22.41	19.10	26.57	19.55	28.14	24.62	26.20	21.47	26.56	25.22	24.08	29.51	30.08	20.49	31.59	21.82

Year and quarter	2019 - Q1		2019 - Q2		2019 - Q3		2019 - Q4		2020 - Q1		2020 - Q2		2020 - Q3		2020 - Q4	
**3. Repeat opioid prescriptions, without a prior visit**																
No. of episodes - age categories	**M**	**F**	**M**	**F**	**M**	**F**	**M**	**F**	**M**	**F**	**M**	**F**	**M**	**F**	**M**	**F**
0-50	82	169	79	146	79	144	70	159	84	161	89	168	88	168	118	132
50-70	134	187	135	166	129	177	147	140	129	108	117	199	114	214	140	212
70+	88	206	84	229	100	226	83	188	89	234	86	184	88	211	85	206
**Total no. of episodes**	304	562	298	541	308	547	300	487	302	503	292	551	290	593	343	550
No. episodes receiving low-value care - age categories	**M**	**F**	**M**	**F**	**M**	**F**	**M**	**F**	**M**	**F**	**M**	**F**	**M**	**F**	**M**	**F**
0-50	30	42	20	40	10	26	11	38	18	38	27	41	15	25	31	19
% low-value	36.59	24.85	25.32	27.40	12.66	18.06	15.71	23.90	21.43	23.60	30.34	24.40	17.05	14.88	26.27	14.39
50-70	29	46	35	35	27	38	39	30	43	28	35	72	33	56	45	59
% low-value	21.64	24.60	25.93	21.08	20.93	21.47	26.53	21.43	33.33	25.93	29.91	36.18	28.95	26.17	32.14	27.83
70+	26	51	27	69	35	60	29	61	36	96	23	70	19	67	20	52
% low-value	29.55	24.76	32.14	30.13	35.00	26.55	34.94	32.45	40.45	41.03	26.74	38.04	21.59	31.75	23.53	25.24
Total no of episodes with low-value treatment	85	139	82	144	72	124	79	129	97	162	85	183	67	148	96	130
% low-value	27.96	24.73	27.52	26.62	23.38	22.67	26.33	26.49	32.12	32.21	29.11	33.21	23.10	24.96	27.99	23.64

Year and quarter	2021 - Q1		2021 - Q2		2021 - Q3		2021 - Q4		2022 - Q1		2022 - Q2		2022 - Q3		2022 - Q4	
**3. Repeat opioid prescriptions, without a prior visit**																
No. of episodes - age categories	**M**	**F**	**M**	**F**	**M**	**F**	**M**	**F**	**M**	**F**	**M**	**F**	**M**	**F**	**M**	**F**
0-50	100	131	100	140	104	158	112	195	146	179	110	185	102	205	129	251
50-70	103	168	104	174	123	238	109	214	97	245	140	231	114	232	124	292
70+	110	202	77	185	96	189	106	216	129	227	129	188	93	193	108	218
**Total no. of episodes**	313	501	281	499	323	585	327	625	372	651	379	604	309	630	361	761
No. episodes receiving low-value care - age categories	**M**	**F**	**M**	**F**	**M**	**F**	**M**	**F**	**M**	**F**	**M**	**F**	**M**	**F**	**M**	**F**
0-50	21	29	18	26	16	43	20	51	33	41	21	41	24	56	29	59
% low-value	21.00	22.14	18.00	18.57	15.38	27.22	17.86	26.15	22.60	22.91	19.09	22.16	23.53	27.32	22.48	23.51
50-70	32	30	20	30	39	44	15	47	19	43	42	66	38	67	27	73
% low-value	31.07	17.86	19.23	17.24	31.71	18.49	13.76	21.96	19.59	17.55	30.00	28.57	33.33	28.88	21.77	25.00
70+	41	59	18	54	35	46	12	66	26	58	34	47	10	43	21	58
% low-value	37.27	29.21	23.38	29.19	36.46	24.34	11.32	30.56	20.16	25.55	26.36	25.00	10.75	22.28	19.44	26.61
Total no of episodes with low-value treatment	94	118	56	110	90	133	47	164	78	142	97	154	72	166	77	190
% low-value	30.03	23.55	19.93	22.04	27.86	22.74	14.37	26.24	20.97	21.81	25.59	25.50	23.30	26.35	21.33	24.97

Additional file 4: separate graphs of both the number of episodes of lower-back and the number of lower-back and knee pain episodes receiving low-value imaging.

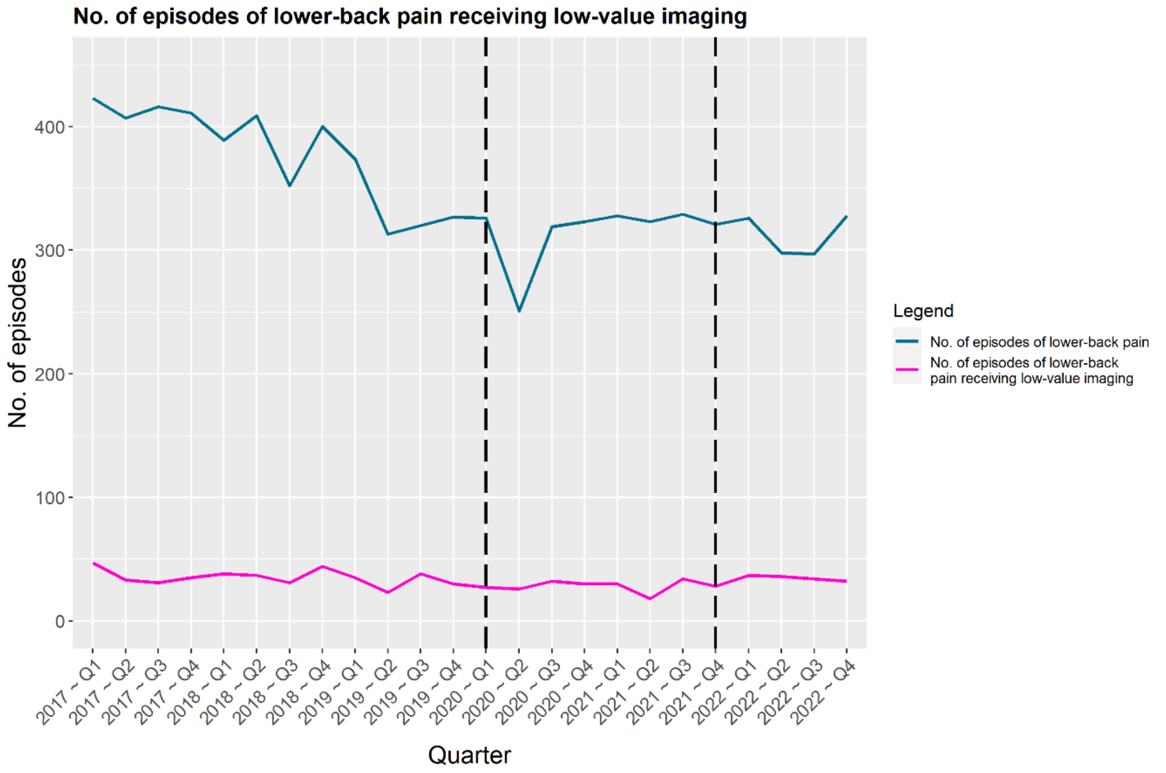

Figure S1: Trends in both the number of episodes of lower-back pain and the number of episodes receiving low-value imaging

Quarter	Total number of episodes of lower-back pain	Total number of episodes of lower-back pain receiving imaging
2017 Q1	423	47
2017 Q2	407	33
2017 Q3	416	31
2017 Q4	411	35
2018 Q1	389	38
2018 Q2	409	37
2018 Q3	352	31
2018 Q4	400	44
2019 Q1	374	35
2019 Q2	313	23
2019 Q3	320	38
2019 Q4	327	30
2020 Q1	326	27
2020 Q2	251	26
2020 Q3	319	32
2020 Q4	323	30
2021 Q1	328	30
2021 Q2	323	18
2021 Q3	329	34
2021 Q4	321	28
2022 Q1	326	37
2022 Q2	298	36

2022 Q3	297	34
2022 Q4	328	32

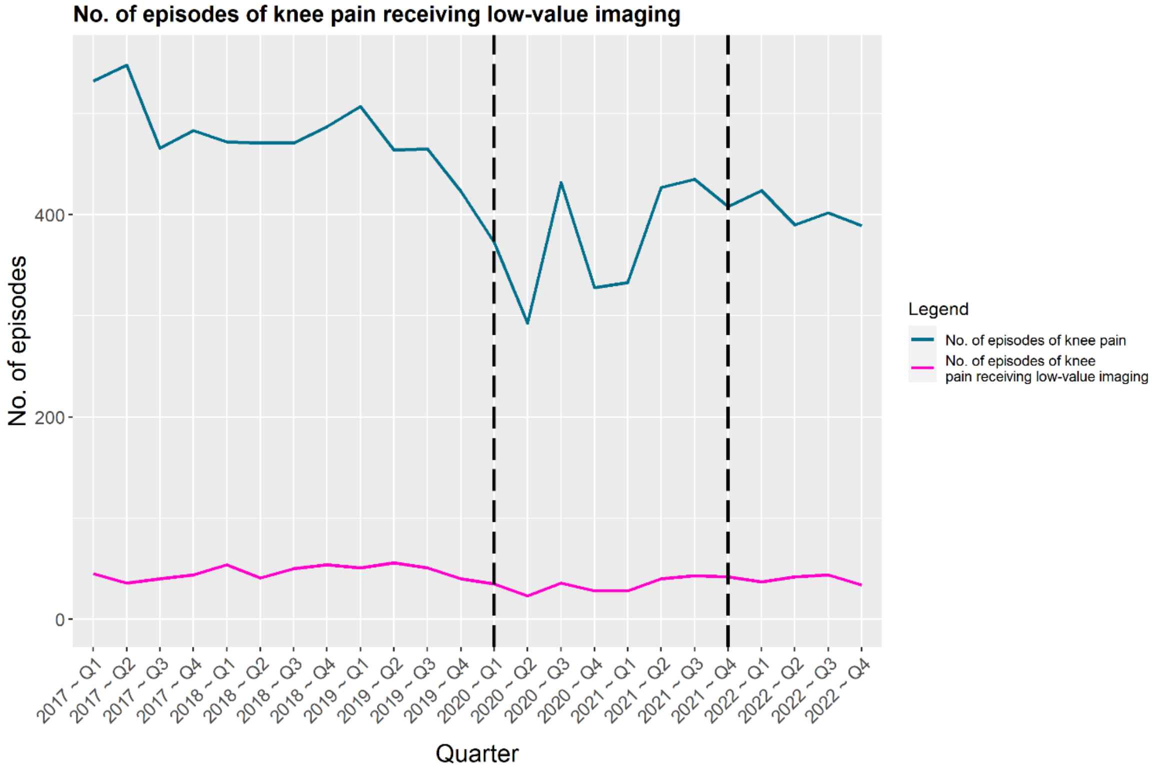

Figure S2: Trends in both the number of episodes of knee pain and the number of episodes receiving low-value imaging

Quarter	Total number of episodes of knee pain	Total number of episodes of knee pain receiving imaging
2017 Q1	532	45
2017 Q2	548	36
2017 Q3	466	40
2017 Q4	483	44
2018 Q1	472	54
2018 Q2	471	41
2018 Q3	471	50
2018 Q4	487	54
2019 Q1	507	51
2019 Q2	464	56
2019 Q3	465	51
2019 Q4	423	40
2020 Q1	373	35
2020 Q2	293	23
2020 Q3	432	36
2020 Q4	328	28
2021 Q1	333	28
2021 Q2	427	40
2021 Q3	435	43
2021 Q4	408	42
2022 Q1	424	37
2022 Q2	390	42

2022 Q3	402	44
2022 Q4	389	34

Additional file 5: Incidence rates of episodes and provision of low-value care for each type of care examined over the examined period corresponding to figure 1. Including rough calculations of the compared incidence rates over the entire periods (e.g. uncorrected for patient characteristics).

1. The use of imaging in the diagnosis of musculoskeletal complaints related to the back or knee.

1. The use of imaging in the diagnosis of musculoskeletal complaints related to the back or knee.					
Year - Quarter	Total number of episodes	Total no. of episodes receiving low-value care	Total no. of patient years (per 1000)	Incidence rate episodes per 1000 patient years	Incidence rate low-value per 1000 patient years
2017 ~ Q1	778	91	2.164	359.52	42.05
2017 ~ Q2	818	71	2.241	365.02	31.68
2017 ~ Q3	735	75	2.303	319.15	32.57
2017 ~ Q4	766	82	2.344	326.79	34.98
2018 ~ Q1	753	95	2.333	322.76	40.72
2018 ~ Q2	781	80	2.396	325.96	33.39
2018 ~ Q3	748	86	2.448	305.56	35.13
2018 ~ Q4	782	99	2.466	317.11	40.15
2019 ~ Q1	779	92	2.314	336.65	39.76
2019 ~ Q2	689	82	2.357	292.32	34.79
2019 ~ Q3	711	90	2.398	296.50	37.53
2019 ~ Q4	707	74	2.420	292.15	30.58
2020 ~ Q1	643	62	2.408	267.03	25.75
2020 ~ Q2	510	51	2.414	211.27	21.13
2020 ~ Q3	719	68	2.442	294.43	27.85
2020 ~ Q4	621	60	2.441	254.40	24.58
2021 ~ Q1	613	59	2.379	257.67	24.80
2021 ~ Q2	726	61	2.402	302.25	25.40
2021 ~ Q3	756	81	2.426	311.62	33.39
2021 ~ Q4	736	78	2.418	304.38	32.26
2022 ~ Q1	738	75	2.351	313.91	31.90
2022 ~ Q2	697	80	2.365	294.71	33.83
2022 ~ Q3	714	82	2.376	300.51	34.51
2022 ~ Q4	745	68	2.355	316.35	28.87

1. The use of imaging in the diagnosis of musculoskeletal complaints related to the back or knee.					
Period	Sum of episodes	Sum of episodes receiving low-value care	Sum of patient years (per 1,000)	Uncorrected - incidence rate episodes over the entire period per 1,000 patient years	Uncorrected - incidence rate low-value over the entire period per 1,000 patient years
**Pre-COVID-19**	9,690	1,079	30.59	316.75	35.27
**COVID-19**	4,681	458	16.92	276.62	27.07
**Post-COVID-19**	2,894	305	9.45	306.34	32.29

2. The prescription of antibiotics for otitis media acuta (OMA) in children without severe symptoms.

2. The prescription of antibiotics for otitis media acuta (OMA) in children without severe symptoms.					
Year - Quarter	Total number of prescriptions	Total no. of low-repeat prescriptions	Total no. of patient years (per 1,000)	Incidence rate prescriptions per 1,000 patient years	Incidence rate low-value per 1,000 patient years
2017 ~ Q1	225	18	0.22	1022.73	81.82
2017 ~ Q2	133	5	0.241	551.87	20.75
2017 ~ Q3	90	10	0.264	340.91	37.88
2017 ~ Q4	143	15	0.286	500.00	52.45
2018 ~ Q1	176	9	0.293	600.68	30.72
2018 ~ Q2	129	6	0.31	416.13	19.35
2018 ~ Q3	106	10	0.327	324.16	30.58
2018 ~ Q4	168	12	0.339	495.58	35.40
2019 ~ Q1	179	10	0.336	532.74	29.76
2019 ~ Q2	112	7	0.35	320.00	20.00
2019 ~ Q3	79	6	0.365	216.44	16.44
2019 ~ Q4	131	6	0.378	346.56	15.87
2020 ~ Q1	223	11	0.385	579.22	28.57
2020 ~ Q2	28	3	0.395	70.89	7.59
2020 ~ Q3	65	3	0.409	158.92	7.33
2020 ~ Q4	61	3	0.417	146.28	7.19
2021 ~ Q1	75	2	0.413	181.60	4.84
2021 ~ Q2	106	1	0.424	250.00	2.36
2021 ~ Q3	165	2	0.438	376.71	4.57
2021 ~ Q4	193	7	0.446	432.74	15.70
2022 ~ Q1	147	3	0.438	335.62	6.85
2022 ~ Q2	244	2	0.443	550.79	4.51
2022 ~ Q3	144	9	0.446	322.87	20.18
2022 ~ Q4	194	9	0.446	434.98	20.18

2. The prescription of antibiotics for otitis media acuta (OMA) in children without severe symptoms.					
Period	Sum of episodes	Sum of episodes receiving low-value care	Sum of patient years (per 1,000)	Uncorrected - incidence rate episodes over the entire period per 1,000 patient years	Uncorrected - incidence rate low-value over the entire period per 1,000 patient years
**Pre-COVID-19**	1,894	125	4.09	462.63	30.53
**COVID-19**	693	21	2.94	235.55	7.14
**Post-COVID-19**	729	23	1.77	411.17	12.97

3. Repeat opioid prescriptions, without a prior visit

3. Repeat opioid prescriptions, without a prior visit					
**Year - Quarter**	**Total number of episodes**	**Total no. of episodes receiving low-value care**	**Total no. of patient years (per 1,000)**	**Incidence rate episodes per 1,000 patient years**	**Incidence rate low-value per 1,000 patient years**
2017 ~ Q1	1,096	225	0.705	1554.61	319.15
2017 ~ Q2	997	224	0.718	1388.58	311.98
2017 ~ Q3	1,023	267	0.731	1399.45	365.25
2017 ~ Q4	1,049	244	0.736	1425.27	331.52
2018 ~ Q1	1,233	317	0.725	1700.69	437.24
2018 ~ Q2	1,080	298	0.736	1467.39	404.89
2018 ~ Q3	1,067	255	0.748	1426.47	340.91
2018 ~ Q4	1,151	296	0.747	1540.83	396.25
2019 ~ Q1	866	224	0.68	1273.53	329.41
2019 ~ Q2	839	226	0.688	1219.48	328.49
2019 ~ Q3	855	196	0.696	1228.45	281.61
2019 ~ Q4	787	208	0.696	1130.75	298.85
2020 ~ Q1	805	259	0.687	1171.76	377.00
2020 ~ Q2	843	268	0.683	1234.26	392.39
2020 ~ Q3	883	215	0.684	1290.94	314.33
2020 ~ Q4	893	226	0.678	1317.11	333.33
2021 ~ Q1	814	212	0.656	1240.85	323.17
2021 ~ Q2	780	166	0.656	1189.02	253.05
2021 ~ Q3	908	223	0.66	1375.76	337.88
2021 ~ Q4	952	211	0.657	1449.01	321.16
2022 ~ Q1	1,023	220	0.637	1605.97	345.37
2022 ~ Q2	983	251	0.637	1543.17	394.03
2022 ~ Q3	939	238	0.634	1481.07	375.39
2022 ~ Q4	1,122	267	0.626	1792.33	426.52

3. Repeat opioid prescriptions, without a prior visit					
**Period**	**Sum of episodes**	**Sum of episodes receiving low-value care**	**Sum of patient years (per 1,000)**	**Uncorrected - incidence rate episodes over the entire period per 1,000 patient years**	**Uncorrected - incidence rate low-value over the entire period per 1,000 patient years**
**Pre-COVID-19**	12,848	3,239	9.29	1382.55	348.54
**COVID-19**	6073	1521	4.67	1299.32	325.42
**Post-COVID-19**	4067	976	2.53	1604.97	385.16
